# Amygdalin based G-6-P synthase inhibitors as novel preservatives for food and pharmaceutical products

**DOI:** 10.1038/s41598-020-70895-1

**Published:** 2020-08-17

**Authors:** Amit Lather, Sunil Sharma, Anurag Khatkar

**Affiliations:** 1 Research Scholar, Faculty of Pharmaceutical Sciences, Maharshi Dayanand University, Rohtak, Haryana India; 2 Department of Pharmaceutical Sciences, G.J.U.S.&T, Hisar, India; 3 Laboratory for Preservation Technology and Enzyme Inhibition Studies, Faculty of Pharmaceutical Sciences, Maharshi Dayanand University, Rohtak, Haryana India

**Keywords:** Screening, Antimicrobial resistance

## Abstract

G-6-P synthase enzyme has been involved in the synthesis of the microbial cell wall, and its inhibition may lead to the antimicrobial effect. In the present study, we designed a library of amygdalin derivatives, and two most active derivatives selected on the basis of various parameters viz*.* dock score, binding energy, and ADMET data using molecular docking software (Schrodinger’s Maestro). The selected derivatives were synthesized and evaluated for their antioxidant and antimicrobial potential against several Gram (+ ve), Gram (−ve), as well as fungal strains. The results indicated that synthesized compounds exhibited good antioxidant, antimicrobial, and better preservative efficacy in food preparation as compared to the standard compounds. No significant differences were observed in different parameters as confirmed by Kruskal–Wallis test (p < 0.05). Docking results have been found in good correlation with experimental wet-lab data. Moreover, the mechanistic insight into the docking poses has also been explored by binding interactions of amygdalin derivative inside the dynamic site of G-6-P synthase.

## Introduction

Nowadays, several antimicrobial and antioxidant based preservatives such as p-hydroxybenzoates, parabens, benzalkonium chloride, dibromodicyanobutane, dimethyl dithiocarbamate, dimethoxy dimethyl hydantoin, formaldehyde, etc. are available in the market^1,2^. However, the existing preservatives have been associated with severe side effects such as estrogenic effect, breast cancer, contact eczema, endocrine disruptors and many other type cancers, etc. Hence, the researchers have been compelled to search for new, better, and safe preservatives^2–5^.

To attain this, the researchers have focused on discovery of novel mechanisms and target sites in addition to the reported mechanisms responsible for antimicrobial action. The different target site for the action of antimicrobials includes inhibition of the cell membrane synthesis, metabolic pathways (folic acid synthesis inhibition), protein synthesis, leakage from cell membrane, and interference with DNA and RNA replication, etc. The microbial cell wall provides mechanical support to microbes and regulates the diffusion process^6^. Hence, for pathogenic bacteria the inhibition of microbial cell wall synthesis may be used as a vital target to produce the antimicrobial effect. The probable target site for the inhibition of microbial cell wall synthesis includes PBPs (transpeptidases), β-lactamase, terminal D-Ala-D-Ala in Lipid II, and Glucosamine-6-Phosphate synthase (G-6-P synthase), etc.^7,8^.

G-6-P synthase is a complex enzyme involved in the formation of Uridine diphosphate acetylglucosamine (UDP-GlcNAc) and catalyzes the first step in hexosamine biosynthesis. It converts Fru-6-P into Glucosamine-6-Phosphate (GlcN-6-P) using glutamine as the source of ammonia. GlcN-6-P is a precursor of Uridine diphosphate N-acetylglucosamine (UDP-NAG) from which other amino sugar-containing molecules are derived. One of these products, N-acetyl glucosamine, is an essential constituent of the peptidoglycan layer of bacterial and fungal cell wall.

The molecular docking can screen thousands of molecules for their affinity towards a particular target site using various softwares^9^. The availability of a three-dimensional crystal structure of G-6-P synthase (protein data bank id 1moq) shall be used to explore, and evaluate a large number of molecules to find out better inhibitors of G-6-P synthase.

A large number of plant-based extracts and phytoconstituents are available, which possess excellent antimicrobial activity; however, there is a lack of data for their preservative effectiveness if compared to the commercially available preservatives^10^. Amygdalin, a cyanogenic glycoside and is present in variable amounts in seeds of fruits like apricot, peach, plum, etc. and fruits like nectarine, chokeberry, christmas berry, barley, brown rice, buckwheat groats, cherry, etc.^11,12^.

The pharmacological potential of amygdalin includes antitussive, antiasthmatic, digestive, anti-atherogenic, inhibition of renal interstitial fibrosis, prevention of pulmonary fibrosis, lung injury due to hypoxia, immune system regulation, antitumor, anti-inflammatory, keratoconjunctivitis sicca, emphysema, leprosy, vitiligo, antimicrobial and antiulcer etc.^13–16^.

Some literature data also concluded that several natural moieties like gallic acid derivatives, ferulic acid, p-coumaric acid, ε-Polylysine, etc. have been evaluated for their preservative effectiveness^17–20^. Hence, based on the available data, it was planned to explore the amygdalin derivatives for their G-6-P synthase inhibitory potential along with their antioxidant, antimicrobial, and preservative efficacy potential in food preparation.

## Experimental

### Material and methods

All the chemicals required for experimental work were of analytical grade and were purchased from LobaChemie, SRL, and Sigma Aldrich. Nutrient agar, nutrient broth, sabouraud dextrose agar, and sabouraud dextrose broth required for antimicrobial and preservative efficacy were obtained from Hi-media Laboratories. Streptomycin, ciprofloxacin, ampicillin and fluconazole were obtained as a gift sample from Belco Pharma, Bahadurgarh. Microbial strains *S. aureus MTCC* 3,160, *P. aeruginosa MTCC* 1934, *E. coli MTCC* 45, *C. albicans MTCC* 183, and *A. niger MTCC* 282 were purchased from MTCC, Chandigarh. Chemical reactions were monitored by TLC on silica gel plates in iodine and UV chamber. The Sonar melting point apparatus in open capillary tube was used for the recording of melting points. ^1^H NMR and ^13^C NMR spectra were confirmed in DMSO and deuterated CDCl_3_ on Bruker Avance II 400 NMR spectrometer at a frequency of 400 MHz downfield to tetramethylsilane standard. The FTIR spectra were recorded on Perkin Elmer FTIR spectrophotometer with the help of the KBr pellets technique. Waters Micromass Q-ToF Micro instrument was used for Mass spectrum recording, and elemental analysis was done by Perkin Elmer 2,400 elemental analyzer.

### In silico molecular docking studies

The Schrodinger, Inc. (New York, USA) software Maestro 11 was used for the computational calculations and docking studies. Laboratory for Enzyme Inhibition Studies, Department of Pharmaceutical Sciences, M.D. University, Rohtak, INDIA, was used for the computational work. The receptor-grid files were generated by grid-receptor generation program Glide, version 6.6, 2015. Grid-based ligand docking was used, which utilized the hierarchical sequence of filters to produce possible conformations of the ligand in the active-site region of the protein receptor. At this stage, raw score values and geometric filters were prepared out unlikely binding modes. The next filter phase involves a grid-based force field evaluation and refinement of docking experiments, including torsional and rigid-body movements of the ligand^21^. The remained docking evaluations were subjected to a Monte Carlo procedure to minimize the energy score.

The energy differences were calculated using the equation:

$$ \Delta E = \, E_{complex} - \, E_{ligand} - \, E_{protein} $$

### Protein preparation

The X-ray protein structure coordinates of G-6-P synthase were downloaded from Protein Data Bank and were prepared with the help of the Schrödinger protein preparation wizard ‘Prepwiz’^22^. Pdb id 1moq, having resolution 1.57 Å was selected based on the lowest resolution and availability. All the water molecules except metals coordinate and present between the ligand and protein were removed. The energy-restrained structure of the protein G-6-P synthase was constructed with the help of the Optimized Potentials for Liquid Simulations -2005 (OPLS-2005) force field.

### Ligand preparation

The three-dimensional structural library was prepared using the Chemdraw software and preceded for energy minimization using the LigPrep tool for the correction of coordinates, ionization, stereochemistry, and tautomeric structure to gain the appropriate conformation through the addition or removal of hydrogen bonds. The partial charges were computed according to the OPLS-2005 force field (32 stereoisomer, tautomers, and ionization) at biological pH and used for molecular docking studies.

### General procedure for the synthesis of amygdalin derivatives

**Figure 1:**
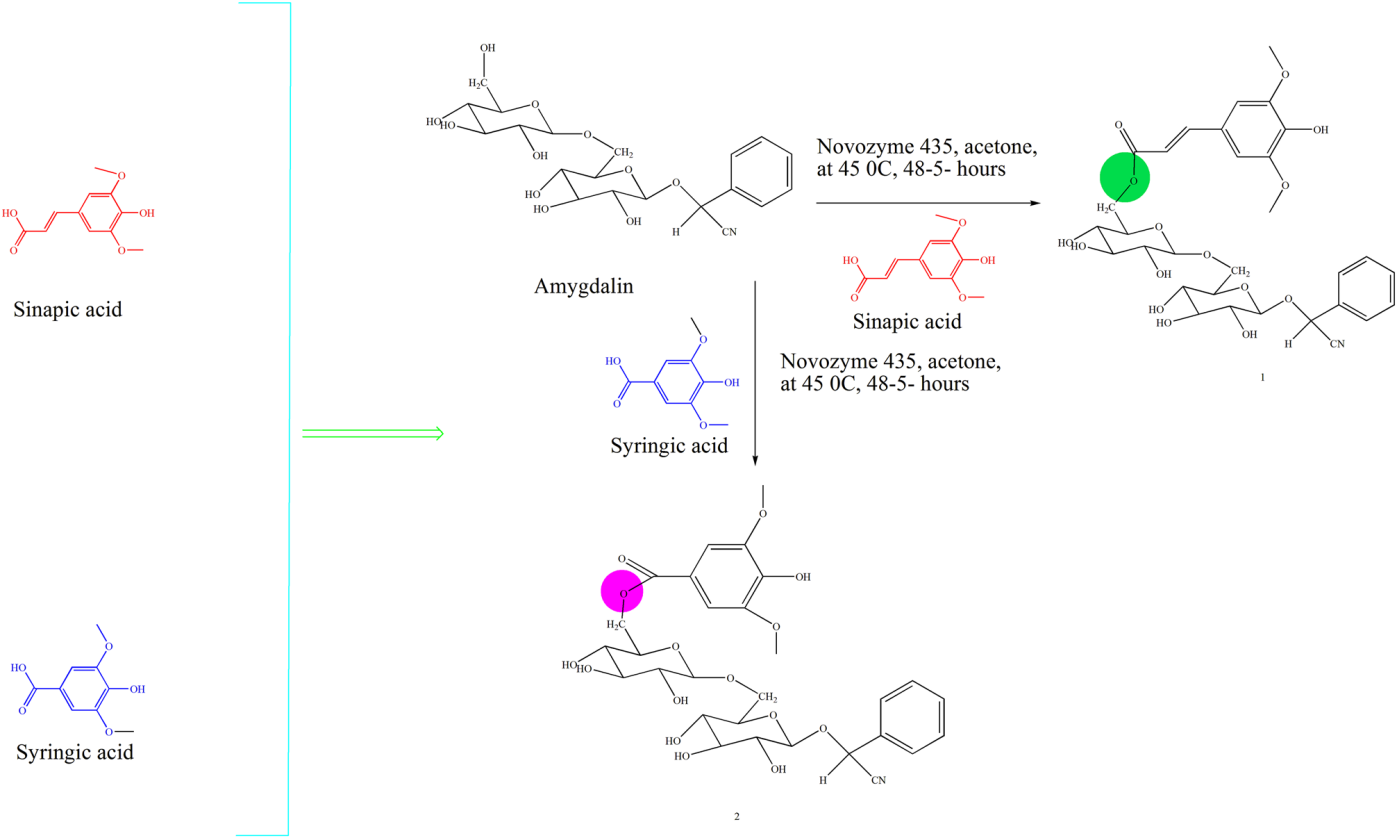
Design strategy and Scheme used for the synthesis of amygdalin derivatives.

The amygdalin derivatives were synthesized by using the procedure outlined in Scheme 1 by enzyme catalysis. Here, 40 ml of acetone containing 0.1 mol/L amygdalin and 0.3 mol/L of sinapic and syringic acid in 1 g of Novozyme 435 were taken in incubator shaker at 200 rpm at 45 °C (48 h). The filtration of the mixture terminated the reaction. Both the compounds in the series were synthesized according to the standard procedures as outlined in Scheme 1 (Fig. 1). The completion of the reaction was confirmed by single spot TLC. After the completion of reaction the concentrated reaction mixture was concentrated and the formed precipitated were filtered off desiccated. The crude products were recrystallized using alcohol yielded compound 1–2. The confirmation of the final compounds was made by physicochemical and spectral methods like FTIR, 1H NMR, 13C NMR spectra, CHN and mass analysis.

### Spectral data

#### (E)-(6-((6-(cyano(phenyl)methoxy)-3,4,5-trihydroxy-tetrahydro-2H-pyran-2yl)methoxy)-3,4,5-trihydroxy-tetrahydro-2H-pyran-2-yl)methyl3-(4-hydroxy-3,5 dimethoxyphenyl) acrylate

mp: 230–232 °C; TLC (Ethyl Acetate: Methanol, 4:1 v/v): R_f_ = 0.60; Yield = 65.50%; M.Wt. = 663.62; ^1^H NMR (400 MHz, CDCL_3_): δ 8.69 (s, 1H), 7.50–7.43 (m, 2H), 7.41–7.32 (m, 3H), 7.32–7.24 (m, 1H), 6.82 (s, 2H), 6.28 (d, J = 15.1 Hz, 1H), 5.80 (s, 1H), 5.00 (d, J = 6.9 Hz, 1H), 4.76 (d, J = 8.8 Hz, 1H), 4.71 (dd, J = 8.8, 2.7 Hz, 3H), 4.59 (d, J = 7.0 Hz, 1H), 4.40–4.30 (m, 2H), 4.33–4.25 (m, 1H), 4.12 (dd, J = 12.5, 7.0 Hz, 1H), 3.90 (dd, J = 12.3, 7.0 Hz, 1H), 3.81 (s, 5H), 3.70–3.56 (m, 2H), 3.56–3.47 (m, 1H), 3.45–3.24 (m, 6H), 3.16 (dt, J = 8.9, 7.0 Hz, 1H); ^13^C NMR (400 MHz, CDCL_3_) δ 168.20, 149.08, 146.99, 138.98, 134.91, 130.91, 130.11, 128.91, 126.93, 119.31, 115.50, 106.56, 104.04, 100.85, 76.13, 74.19, 74.10, 74.00, 73.12, 72.60, 71.10, 70.23, 68.91, 68.25, 63.67, 56.59; IR (KBr pellets): 1,029 cm^−1^ (–C–O–C), 1,074 cm^−1^ (–C–C–), 1,449 cm^−1^ (–C=C–), 1642 cm^−1^ (–C=N–), 2,875 cm^−1^ (–C–H–), 3,371 (–OH–); MS ES + (ToF): m/z 663.22 [M^+^ + 2]; CHNS: Calc (C_31_H_37_NO_15_): C, 56.11; H, 5.62; N, 2.11; O, 36.16; Found C, 56.14; H, 5.64; N, 2.10; O, 36.18.

#### (6-((6-(cyano(phenyl)methoxy)-3,4,5-trihydroxy-tetrahydro-2H-pyran-2-yl)methoxy)-3,4,5-trihydroxy-tetrahydro-2H-pyran-2-yl)methyl-4-hydroxy-3,5 dimethoxy benzoate

mp: 239–241 °C; TLC (Ethyl Acetate: Methanol, 4:1 v/v): R_f_ = 0.67; Yield = 69.55%; M.Wt. = 622.55; ^1^H NMR (400 MHz, CDCL_3_): δ 8.69 (s, 1H), 7.50–7.43 (m, 2H), 7.41–7.32 (m, 2H), 7.32–7.24 (m, 1H), 7.18 (s, 2H), 5.80 (s, 1H), 5.00 (d, J = 6.9 Hz, 1H), 4.76 (d, J = 8.8 Hz, 1H), 4.71 (dd, J = 8.8, 2.6 Hz, 3H), 4.59 (d, J = 7.0 Hz, 1H), 4.37 (d, J = 8.0 Hz, 1H), 4.33–4.25 (m, 1H), 4.24 (dd, J = 12.4, 7.0 Hz, 1H), 4.01 (dd, J = 12.4, 7.0 Hz, 1H), 3.90 (dd, J = 12.3, 7.0 Hz, 1H), 3.81 (s, 5H), 3.65 (dd, J = 12.3, 6.9 Hz, 1H), 3.57–3.27 (m, 7H), 3.16 (dt, J = 8.9, 6.9 Hz, 1H)); ^13^C NMR (400 MHz, CDCL_3_): δ 166.71, 147.85, 141.19, 134.88, 130.91, 130.11, 128.91, 120.59, 119.27, 106.79, 102.61, 101.58, 76.55, 75.99, 75.68, 75.49, 74.47, 73.99, 71.60, 70.81, 69.52, 68.85, 64.07, 56.35; IR (KBr pellets): 1,029 cm^−1^ (–C–O–C), 1,074 cm^−1^ (–C–C–), 1,449 cm^−1^ (–C=C–), 1642 cm^−1^ (–C=N–), 3,029 cm^−1^ (–C–H–), 3,371 cm^−1^ (–OH–); MS ES + (ToF): m/z 622.18 [M^+^ + 2]; CHNS: Calc (C_28_H_32_NO_15_): C, 54.02; H, 5.18; N, 2.25; O, 38.55; Found C, 54.05; H, 5.15; N, 2.27; O, 38.56.

### Antioxidant activity

#### 2,2-Diphenyl-1-pycrilhydrazil hydrate (DPPH) radical scavenging assay

Antioxidant activity of the synthesized was evaluated photocolorimetric assay by using DPPH free radical scavenging method. Briefly, 0.1 mM solution of DPPH in methyl alcohol was prepared, and 1 mL of this solution was added to 3 mL of sample or standard. Discolorations were measured at 517 nm after incubation for 30 min at 30 °C in the dark. Lower absorbance of the reaction mixture indicates higher free radical scavenging activity. The test was performed in triplicate and the % inhibition values of given samples was calculated by using the formula:

$$ \% {\text{ Inhibition }} = \, \left( {{\text{A}}_{{\text{c}}} - {\text{A}}_{{\text{s}}} } \right) \, \times { 1}00/{\text{A}}_{{\text{c}}} $$

Here, A_c_ was the absorbance of the control, and A_s_ was the absorbance of the sample^23^.

### Antimicrobial activity

#### Minimum inhibitory concentration (MIC)

The antimicrobial activity of the synthesized compounds was determined against *S. aureus MTCC* 3,160, *P. aeruginosa MTCC* 1934, *E. coli MTCC* 45, *P. mirabilis MTCC* 3,310*, C. albicans MTCC* 183, and *A. niger MTCC* 282 by using the tube dilution method. Dilutions of test and standard compounds were prepared in double strength nutrient broth I.P. (bacteria) or sabouraud dextrose broth I.P. (fungi)^24^. The slants of *E. coli*, *P. aeruginosa, P. mirabilis,* and *S. aureus* were incubated at the 30–35 °C for 24 h. The slants of *C. albicans* were incubated at 20–25 °C for 48 h, whereas; the slants of *A. niger* were incubated at 20–25 °C for 5 days. After the incubation period sterilized 0.9% NaCl solution was used to harvest the bacterial, and fungal cultures from agar slant through proper shaking and then the suspensions of microorganisms were diluted with the sterile 0.9% NaCl solution to Colony Forming Unit (CFU) count was adjusted by adjusting the density of microorganism suspension to that of 0.5 McFarland standards by adding distilled water. The number of CFU was determined by dilution pour-plate method. A serial dilution of 50 µg/mL, 25 µg/mL, 12.5 µg/mL, 6.25 µg/mL, 3.12 µg/mL and 1.62 µg/mL was used for determination of MIC. The samples tubes were incubated at 37 °C for 24 h (bacteria), at 25 °C for 7 days (*A. niger*), and at 37 °C for 48 h (*C. albicans*), and the results were recorded in pMIC^25^.

#### Preservative effectiveness study

The selected most active antioxidant/antimicrobial compounds were further evaluated for their preservative potential in the cosmetic product, White lotion USP and food products; such as fresh aloe vera juice the cosmetic product as per the procedure mentioned in USP 2004^26^.

#### Preparation of aloe vera juice

Aloe vera leaves were cleaned with distilled water and cleaned with 0.5% chlorine water. The leaf base and tip were chopped 1.5 inches and 3 inches, respectively. Margins of leaves were removed with the help of a stainless steel knife. The pulp was washed 2–3 times with distilled water to remove the exudates and homogenized with the help of a blender, and then filtered through an autoclave muslin cloth. The aloe vera juice thus obtained was used for the testing of food preservative efficacy^27^.

#### Preparation of white lotion USP

Ingredients: Zinc sulfate 40 gm, sulfurated potash 40 gm and purified water q.s. to 1,000 mL. Firstly, zinc sulfate and sulfurated potash were dissolved in 450 mL of water separately and filtered. Then, sulfurated potash solution was added to zinc sulfate with stirring. At last, the required amount of water was added and mixed thoroughly and sterilized. For preservative efficacy testing, the White lotion USP was prepared using the equimolar amount of compounds **1**–**2** as novel preservatives by replacing sodium benzoate, methyl paraben and propyl paraben from the formula^28^.

#### Challenge microorganism

*S. aureus MTCC* 3,160, *P. aeruginosa MTCC* 1934, *E. coli MTCC* 45, *C. albicans MTCC* 183, and *A. niger MTCC* 282 were used as common contaminants in the study as prescribed in USP for preservative efficacy testing in the pharmaceutical preparations^29^.

#### Preparation of inoculums

The slants of *E. coli*, *P. aeruginosa,* and *S. aureus* were incubated at the 37 °C for 24 h. The slants of *C. albicans* were incubated at 37 °C for 48 h, whereas; the slants of *A. niger* were incubated at 25 °C for 7 days^29^.

### Preservative efficacy procedure

#### Aloe vera juice

Preservative efficacy of the selected compound 1 and sodium benzoate (standard) was estimated in freshly prepared aloe vera juice as per the method with minor modifications as described by Ahlawat et al*.*^30^. A concentration of 600 mg/kg or 600 ppm of sodium benzoate in aloe vera juice was taken as per food safety and standard guidelines. Equimolar quantity (0.0004 mol) of selected compound was taken as a preservative system in test samples. Challenged microbial cell suspension was inoculated the juice preparation with inoculum size never exceed more than 1%. After inoculation with microbes juice was incubated at room temperature for consecutive 28 days, and samples were collected on the 14th and 28th day of the experiment. The viable count of microorganisms was performed on nutrient agar (bacteria), and sabouraud dextrose agar (fungi) plates^31^. Each experiment was done in triplicate and log cfu/ml of juice was determined with comparison to standard.

#### White lotions USP

White lotions USP was added in final containers and were used in the challenge test. The preparation was inoculated with a 0.5–1% volume of microbial inoculum having a concentration of 1 × 10^5^–1 × 10^6^ CFU/mL^32^. Inoculated samples were mixed thoroughly to ensure homogeneous microorganism distribution and incubated. The CFU/mL of the product was determined at an interval of 0 days, 7 days, 14 days, 21 days, and 28 days in agar plates. Log CFU/mL of white lotion USP was calculated as not as not less than 2.0 log reductions from initial count on 14th day of incubation and no increase in CFU from 14th day count to 28th day in case of bacteria and no increase from the initial calculated count on 14th day and 28th day in case of fungi^33^.

#### Stability study

Compound 1 was selected for stability study in their final container containing the formulation of Aole vera gel and White Lotion USP. Formulation having different preservatives i.e., standard and compound **1** were stored at 40° ± 2 °C at 75% RH ± 5% RH (as per ICH guidelines) and were analyzed for the pH and cfu/ml of the product at 0, 1, 2, 3, 4, 5, and 6 months.

### Statistical analyses

Data are expressed as mean values ± standard deviation or standard error as described in the legend of the figures and tables. Analysis of results was done by Kruskal–Wallis test as appropriate. Differences were considered to be statistically significant at P < 0.05. Statistical analyses were performed using MS excel data statistics analysis tool.

## Results and discussion

### Molecular docking study

**Figure 2:**
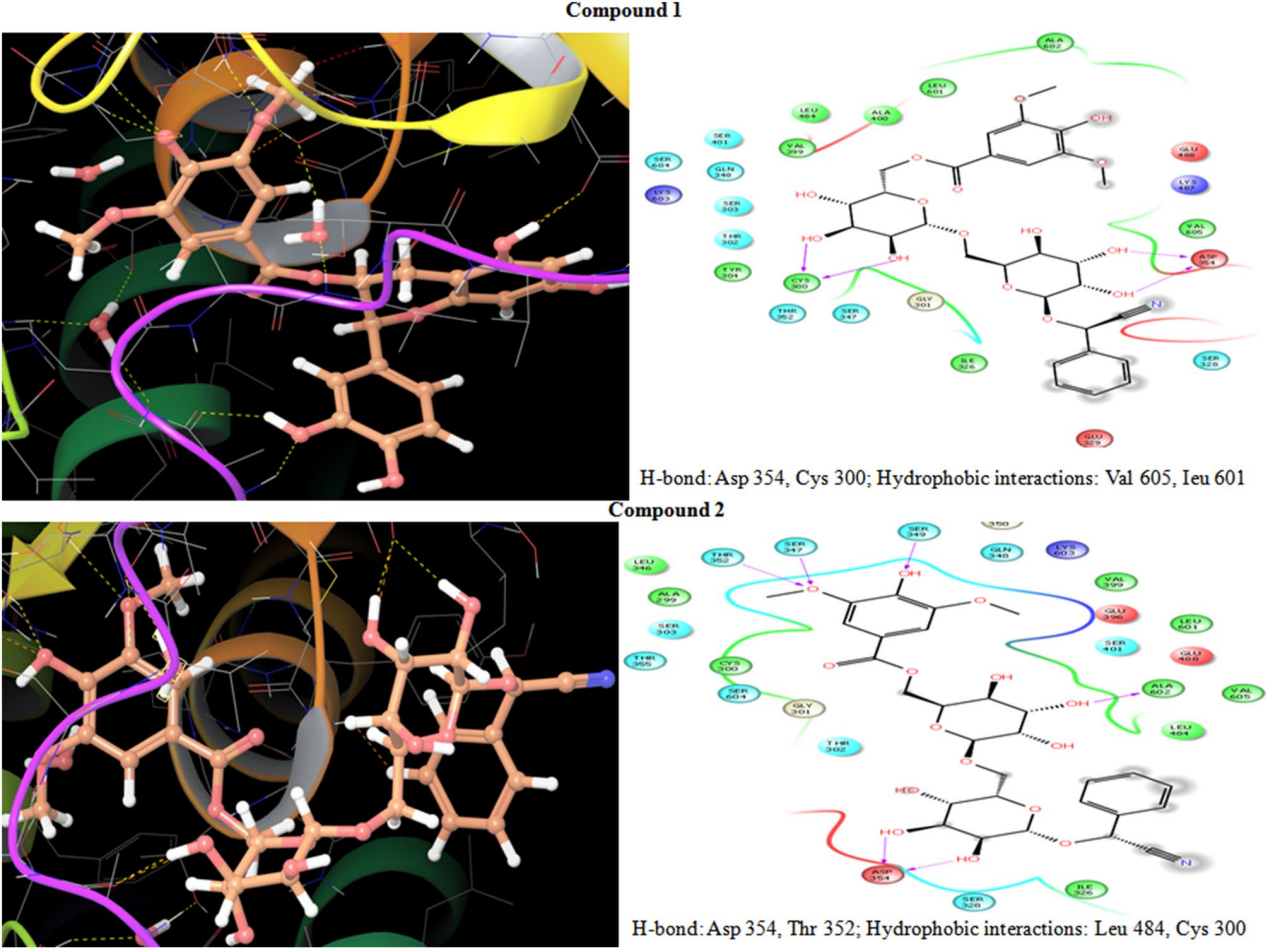
Interaction patterns of ligands within the G-6-P synthase pocket.

The selection of amygdalin for further evaluation as preservative has been made on the basis of our prior evaluation in docking and absorption, distribution, metabolism and excretion (ADMET) study data^34^. Then the proposed library of amygdalin derivatives was again evaluated for their molecular docking behavior with the help of Schrodinger’s Maestro docking software^35^. Molecules were docked with the help of the G-6-P synthase crystallographic complex having pdb id 1moq. The Induced Fit docking (IFD) method of docking was utilized for the same. The predicted binding pattern revealed that synthesized ligand binds within the catalytic cavity of G-6-P synthase firmly via hydrogen bond formation, pi-pi stacking, and hydrophobic interactions. Two compounds 1 and 2, were selected via docking score, binding energy, and ADMET study parameters. Compound 1 showed the hydrogen bonding with Asp 354, Cys 300, and hydrophobic interactions with Val 605, Leu 601, while compound 2 showed hydrogen bonding with Asp 354, Ala 602, Ser 349, Ser 347, Thr 352, and hydrophobic interactions Leu 484, Cys 300. The synthesized compounds 1 and 2 possessed excellent dock score − 9.65, − 6.97, respectively and binding energy − 1.40 kJ/mol, − 51.31 kJ/mol, respectively as compared to standard drugs ciprofloxacin, ampicillin and fluconazole, dock score − 5.18, − 5.06, − 5.12, and binding energy − 37.16 kJ/mol, − 25.41 kJ/mol and − 23.15 kJ/mol, respectively. Hence, it is cleared that both the compounds 1 and 2 behave as G-6-P synthase inhibitor. Here, the inhibition of G-6-P synthase enzyme further evaluated by the outcomes of the inhibition likes antimicrobial activity. This further made the clearance behind the inhibition of G-6-P synthase enzyme by different proposed molecules. The molecular docking of the proposed amygdalin derivatives with the target site of G-6-P synthase (PDB ID 1MOQ) showed that all the selected compounds exhibited better binding affinity with different amino acid residues in active pocket of the enzyme. The results of molecular docking for different ligands within G-6-P synthase pocket and their interaction with different amino acid residues have been shown in Fig. 2. Molecular docking results of proposed amygdalin derivatives have been shown in Table 1.

**Table 1:** Docking parameters, ADMET profile and pMIC value of selected amygdalin derivatives.

Compound(s)	G-6-P synthase binding affinity		ADMET profile							pMIC values in μM						
	Docking score	Energy	No. of rotatable bond	DonorHB	AcceptHB	QplogPo/w	QplogBB	QPPMDCK	QPPCaco	*K.pneumoniae*	*P. mirabilis*	*P. aeruginosa*	*S. aureus*	*E. coli*	*C.albicans*	*A. niger*
Compound 1	− 9.65	− 71.40	8	6	20	− 2.01	− 4.41	0.84	2.70	2.02	1.72	1.42	1.42	2.02	1.72	2.02
Compound 2	− 6.97	− 51.31	10	7	22	− 1.97	− 4.34	0.92	3.0	1.39	1.39	1.69	1.39	1.69	1.39	1.39
Amygdalin	− 6.60	− 57.22	6	5	18	− 1.02	− 3.35	0.98	4	0.86	1.06	1.76	1.06	0.96	0.76	0.86
Streptomycin	− 5.44	− 40.20	9	12	15	− 2.06	− 4.20	0.78	3	1.96	1.06	1.36	1.06	1.96	–	–
Ciprofloxacin	− 5.18	− 37.16	3	2	6	− 1.02	2.23	0.80	4	2.02	1.12	1.42	1.12	1.42	–	–
Ampicillin	− 5.06	− 25.41	4	3	5	− 1.35	0.99	0.90	0.89	2.04	1.14	0.84	0.84	1.74	–	–
Fluconazole	− 5.12	− 23.15	5	1	5	− 2.32	0.88	0.87	0.93	–	–	–	–	–	1.08	1.38

### ADMET study

The compounds selected from docking have been further evaluated for their ADME parameters so that the selection of final preservative becomes easy. The evaluation of different ADMET parameters of selected amygdalin derivatives has been represented in Table 1. All the synthesized compounds fulfilled the standard Rule of Five^36^. All the synthesized compounds qualified the conditions for various descriptors like lipophilicity (LogP), hydrogen bond acceptor (HBA), hydrogen bond donor (HBD) and moleculat weight (MW). All parameters were in a suitable range for drug-like characteristics. In addition, according to Veber et al*.* (2002) for better bioavailability, rotatable bonds should be ≤ 10 as the rotatable bonds in ligand impart elasticity^37^. The values of QPlogBB should be > 1.0 CNS active compounds, and value < 1.0 CNS inactive compounds. QPPCaco cell permeability should be in a range from 4–70^38^. All the synthesized compounds exhibited a suitable drug-like profile and could be used for further evaluation as a novel preservative for food and pharmaceutical preparations.

### Chemistry

The amygdalin derivatives selected on the basis of docking and ADMET parameters were synthesized **(**derivatives 1–2) by the reaction according to Vemula et al*.* (2006), outlined in Scheme 1^39^. The chemical structural parts of all the synthesized compounds were confirmed by FTIR, ^1^H NMR, ^13^C NMR, mass spectroscopy, and elemental analysis, which were in full agreement with their structures. The synthesis of amygdalin esters was completed by enzyme catalysis. In general, 40 ml of acetone containing 0.1 mol/L amygdalin and 0.3 mol/L vinyl ester was added in 1 g of Novozyme 435. The reaction mixtures were placed it in an incubator shaker at 200 rpm on 45 °C for 48 h. The filtration of reaction mixtures terminated the reaction.

Both of the compounds were synthesized according to the standard procedures as outlined in Scheme 1. The completion of the reaction was confirmed by TLC under UV lamp and FTIR. Formation of compounds 1 and 2 was further confirmed by peak shifted from 2,730 cm^−1^ (–OH) and appearance of a peak at 1,730 cm^−1^ and 1744 cm^−1^ (–C=O), respectively for compound 1 and compound 2. The change in chemical shift value, coupling constant and multiplicities were analyzed by ^1^HNMR and ^13^C NMR signals of synthesized compounds. The FTIR, ^1^H NMR and ^13^C NMR data confirmed the chemical structures of synthesized amygdalin derivatives. Final confirmation of the synthesized compounds was done by analyzing the mass spectrum of synthesized derivatives for molecular weight determination, and the Q-ToF Micro instrument was used as ion source. Most of the derivatives showed M^+^ (molecular ion peak), (M^++1^), (M^++2^) in positive chemical ionization, and (M^1+^), (M^2+^), M^+^ during negative chemical ionization mode. Finally, establishment of synthesis of amygdalin derivatives was done by elemental analysis where C, H, and N in percent were found within acceptable limits.

### Antioxidant activity

#### DPPH radical scavenging activity

The plant-based antioxidants can be used in food and pharmaceuticals to enhance their shelf life against oxidation^40^. In the present study, DPPH radical scavenging assay method was utilized for the evaluation of the antioxidant profile of the synthesized compounds^41^. In this screening, compound 1 was observed as the most potent antioxidant compound (IC_50_ values 5.54 ± 0.03 µM) as compared to reference standard L-ascorbic acid (IC_50_ values 8.11 ± 0.0.69 µM). However, compound 2 showed moderate antioxidant activity (IC_50_ value 6.51 ± 0.04 µM). The antioxidant activity of the amygdalin was found 7.72 ± 0.03 µM^42^. Here, the better antioxidant property of amygdalin derivative shall be useful in the preservation of food, cosmetics, and pharmaceuticals^43^. All the results were expressed as mean ± standard deviation (n = 5) and results were found significant with Krukal-Wallis test (p < 0.05).

### Antimicrobial activity

#### MIC

Newly synthesized amygdalin derivatives were evaluated for their in vitro antimicrobial activity against standard MTCC strains of *K. reparati, P. mirabilis, P.aeruginosa, S. aureus, E. coli, C. albicans,* and *A. niger*. Antimicrobial activity of the test compounds revealed that the compound **1** was found to be the most potent compound ((pMIC 2.02, 1.72, 1.42, 1.42, 2.02, 1.72 and 2.02 µM/ml against *P. mirabilis, P. aeruginosa*, *S. aureus, E. coli, C. albicans,* and *A. niger* respectively) as compared to the standard drugs streptomycin (pMIC 1.06, 1.36, 1.06, and 1.96 μM for *P. mirabilis, P. aeruginosa, S. aureus,* and *E. coli* respectively), ciprofloxacin (pMIC 1.12, 1.42, 1.12, and 1.42 μM for *P. mirabilis, P. aeruginosa, S. aureus,* and *E. coli* respectively), ampicillin (pMIC 1.14, 0.84, 0.84, and 1.74 μM for *P. mirabilis, P. aeruginosa, S. aureus,* and *E. coli* respectively) and fluconazole (pMIC 1.08 and 1.38 μM for *C. albicans,* and *A. niger* respectively) using tube dilution method. Here, the results of MIC studies (Table 1) revealed that the synthesized compounds have better antimicrobial potential as compared to standard ciprofloxacin, ampecillin, and fluconazole. The probable mechanism of antimicrobial activity of amygdalin derivatives may be due to the better inhibition of G-6-P synthase.

#### Preservative efficacy

The results of preservative efficacy study of the aloe vera juice and White lotion USP were performed and were reported as mean ± standard deviation. Results of microbial growth at 14th day and 28th day were found to be significant with p < 0.05 as confirmed by Kruskal–Wallis test.

#### Aloe vera juice

**Figure 3:**
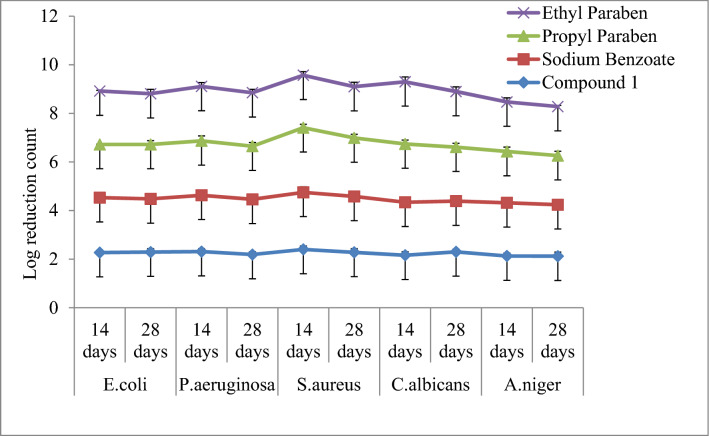
Preservative efficacy results of compound 1 in Aloe vera juice and degree of microbial log reduction. Data are means of five replicates; standard deviation is shown as error bars. Chart indicates statistically significant differences between groups (p < 0.05).

The results of preservative effectiveness have been summarized in Table 2. The log CFU/ml for compound 1 revealed that the values were within the prescribed limit as per USP criteria. The selected compound 1 reduced the growth of microbes on the 14th day from the initial count and found to be effective on the 28th day and results were also comparable to sodium benzoate. The preservative efficacy of the amygdalin compound 1 has been represented for number of days vs. degree of microbial log reduction and has been shown graphically in Fig. 3.

**Table 2:** Log CFU/ml values of the selected compound **1** in Aloe vera juice and White lotion USP.

Compound		*E.coli*		*P.aeruginosa*		*S.aureus*		*C.albicans*		*A.niger*	
Cfu/ml after days		14 days	28 days	14 days	28 days	14 days	28 days	14 days	28 days	14 days	28 days
Compound 1	#	2.25 ± 0.10^a^	2.19 ± 0.14^b^	2.31 ± 0.12^c^	2.19 ± 0.12^d^	2.4 ± 0.14^e^	2.28 ± 0.15f.	2.16 ± 0.15^g^	2.3 ± 0.11^h^	2.13 ± 0.12^i^	2.12 ± 0.17^j^
	@	3.22 ± 0.12^a^	3.20 ± 0.13^b^	3.31 ± 0.12^c^	3.22 ± 0.22^d^	3.11 ± 0.14^e^	3.62 ± 0.13f.	3.22 ± 0.16^g^	3.12 ± 0.21^h^	3.14 ± 0.22^i^	3.64 ± 0.12^j^
Sodium Benzoate	#	2.26 ± 0.11^a^	2.19 ± 0.12^b^	2.32 ± 0.13^c^	2.27 ± 0.22^d^	2.35 ± 0.23^e^	2.3 ± 0.21f.	2.18 ± 0.16^g^	2.09 ± 0.18^h^	2.19 ± 0.17^i^	2.12 ± 0.15^j^
	@	3.13 ± 0.21^a^	3.23 ± 0.22^b^	3.21 ± 0.16^c^	3.22 ± 0.13^d^	3.54 ± 0.24^e^	3.26 ± 0.12f.	3.17 ± 0.08^g^	2.22 ± 0.28^h^	3.17 ± 0.13^i^	3.13 ± 0.12^j^
Propyl Paraben	#	2.19 ± 0.15^a^	2.24 ± 0.16^b^	2.24 ± 0.2^c^	2.19 ± 0.15^d^	2.66 ± 0.14^e^	2.41 ± 0.15f.	2.4 ± 0.16^g^	2.22 ± 0.16^h^	2.11 ± 0.18^i^	2.02 ± 0.18^j^
	@	3.22 ± 0.51^a^	3.22 ± 0.26^b^	3.32 ± 0.34^c^	3.33 ± 0.16^d^	3.23 ± 0.22^e^	3.22 ± 0.23f.	3.29 ± 0.13^g^	3.22 ± 0.21^h^	3.19 ± 0.16^i^	3.10 ± 0.18^j^
Ethyl Paraben	#	2.2 ± 0.18^a^	2.09 ± 0.18^b^	2.24 ± 0.16^c^	2.22 ± 0.14^d^	2.16 ± 0.15^e^	2.11.18f.	2.56 ± 0.2^g^	2.29 ± 0.19^h^	2.04 ± 0.17^i^	2.02 ± 0.12^j^
	@	3.39 ± 0.12^a^	3.10 ± 0.17^b^	3.23 ± 0.36^c^	3.39 ± 0.12^d^	3.12 ± 0.13^e^	3.19 ± 0.12f.	3.50 ± 0.21^g^	3.20 ± 0.41^h^	3.17 ± 0.54^i^	3.09 ± 0.28^j^
Control	**5 ± 0.20**										

^#^ Aloe vera juice; ^@^White lotion USP.

*CFU* Colony forming unit, all experiments were conducted in triplicate (n = 5) and the mean values are presented. Different letters mean p < 0.05 in each line by Kruskal–Wallis test.

#### White lotion USP

**Figure 4:**
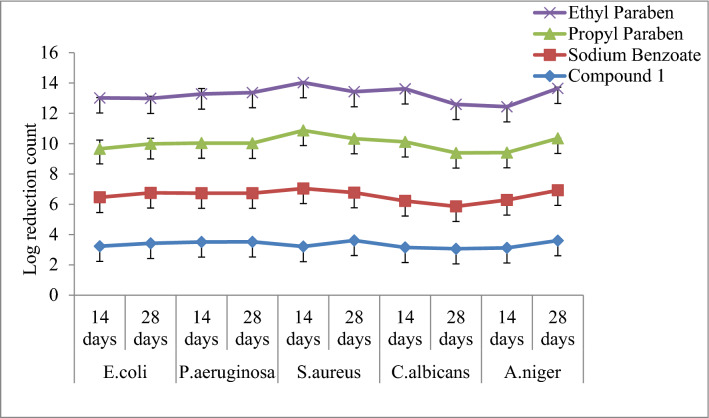
Preservative efficacy results of compound 1 in White Lotion USP and degree of microbial log reduction. Data are means of five replicates; standard deviation is shown as error bars. Chart indicates statistically significant differences between groups (p < 0.05).

The highly active antimicrobial compound 1 was selected for evaluation of its preservative efficacy. Result showed a less than 2.0 log reductions from initial count on 14th day and number of CFU/ml in some samples increased on the 14th day to 28th day as compared to that of the standard preservatives sodium benzoate, propyl paraben and ethyl paraben. The log CFU/ml (Table 2) for compound 1 revealed that the values were within the prescribed limit as per USP criteria^28^. The graphical representation of preservative efficacy of amygdalin compound 1 in white lotion USP has been presented between the numbers of days *vs*. degree of microbial log reduction and graphically same has been represented in Fig. 4.

#### Stability study

The results of stability testing were performed in triplicate, and were reported as mean values in Table 3. Results revealed that the pH of Aloe vera juice and White lotion USP samples were in range of 7.6–8.0, which indicated the stability of compound 1 ((E)-(6-((6-(cyano(phenyl)methoxy)-3,4,5-trihydroxy-tetrahydro-2H-pyran-2yl)methoxy)-3,4,5-trihydroxy-tetrahydro-2H-pyran-2-yl)methyl3-(4-hydroxy-3,5 dimethoxyphenyl) acrylate) as preservative over, the six months period as compared to that of the standard preservatives sodium benzoate, propyl paraben, and methyl paraben. The results of the microbial study indicated that no microbial growth was observed in samples containing compound **1,** over 6 months period. These results indicated that the products were found stable over 6 months with added preservatives. Results for microbial growth and pH changes also found to be significant at p < 0.05.

**Table 3:** Stability studies of compound **1 **in Aloe vera juice and White Lotion USP for pH.

Compound(s)		Change in pH with time						
		0 month	1 month	2 month	3 month	4 month	5 month	6 month
Compound 1	#	7.9 ± 0.32^a^	8.2 ± 0.34^b^	7.4 ± 0.14^c^	7.8 ± 0.24^d^	7.3 ± 0.30^e^	7.8 ± 0.22f.	7.6 ± 0.34^g^
	@	7.8 ± 0.25^a^	7.8 ± 0.22^b^	7.9 ± 0.33^c^	7.7 ± 0.33^d^	7.6 ± 0.32^e^	7.7 ± 0.22f.	8.1 ± 0.23^g^
Sodium benzoate	#	8.8 ± 0.14^a^	9.2 ± 0.39^b^	9.2 ± 0.21^c^	9.4 ± 0.39^d^	9.1 ± 0.42^e^	9.2 ± 0.22f.	9.2 ± 0.56^g^
	@	9.2 ± 0.54^a^	9.2 ± 0.84^b^	9.4 ± 0.33^c^	9.7 ± 0.43^d^	9.2 ± 0.50^e^	9.1 ± 0.94f.	9.2 ± 0.17^g^
Propyl paraben	#	7.3 ± 0.21^a^	7.5 ± 0.25^b^	7.5 ± 0.25^c^	7.5 ± 0.25^d^	7.8 ± 0.28^e^	7.3 ± 0.33f.	7.2 ± 0.54^g^
	@	8.2 ± 0.04^a^	8.5 ± 0.69^b^	8.8 ± 0.68^c^	8.7 ± 0.76^d^	8.5 ± 0.32^e^	8.3 ± 0.39f.	8.7 ± 0.26^g^
Ethyl paraben	#	8.2 ± 0.02^a^	8.4 ± 0.44^b^	8.4 ± 0.26^c^	8.5 ± 0.24^d^	8.4 ± 0.21^e^	8.3 ± 0.49f.	8.4 ± 0.28^g^
	@	8.4 ± 0.35^a^	8.6 ± 0.36^b^	8.0 ± 0.66^c^	8.2 ± 0.18^d^	8.1 ± 0.14^e^	8.1 ± 0.69f.	8.4 ± 0.32^g^
Control	# 8.0 ± 23; @ 8.2 ± 0.08							

^#^Aloe vera juice; ^@^White lotion USP.

All pH values were recorded in triplicate (n = 5) and the mean values are presented. Different letters mean p < 0.05 in each line by Kruskal–Wallis test.

## Conclusion

It has also been reported in our previous study that amygdalin can act as an active inhibitor of G-6-P synthase enzyme based upon the results of molecular docking and ADMET data^43^. In current study the amygdalin derivatives were found active G-6-P synthase inhibitors, and molecular docking study, provided a new insight of mechanism for the inhibition with visual binding interactions. The derivatives of amygdalin ((E)-(6-((6-(cyano(phenyl)methoxy)-3,4,5-trihydroxy-tetrahydro-2H-pyran-2yl)methoxy)-3,4,5-trihydroxy-tetrahydro-2H-pyran-2-yl)methyl3-(4-hydroxy-3,5 dimethoxyphenyl) acrylate) showed antioxidant, antimicrobial, better preservative efficacy and prevent the change in pH as well microbial count of formulation for food as well as pharmaceutical products, which were in agreement with the results of molecular docking and highlight the mechanism of their preservative activity. Therefore, the synthesized amygdalin derivatives can be used as novel food and pharmaceutical preservatives to prevent them from microbial degradation.

## Data Availability

The datasets used and/or analyzed during the current study are available from the corresponding author on reasonable request.
